# Enhancing the Efficiency of Resilient Multipath-Routed Elastic Optical Networks: A Novel Approach for Coexisting Protected and Unprotected Services with Idle Slot Reuse

**DOI:** 10.3390/s24123965

**Published:** 2024-06-19

**Authors:** Michael M. L. Cavalcanti, Gabriela W. Teixeira, Henrique A. Dinarte, Raul C. Almeida, Raouf Boutaba, Daniel A. R. Chaves

**Affiliations:** 1 Polytechnic School of Pernambuco (Poli), University of Pernambuco (UPE), Recife 50720-001, PE, Brazil; 2 Department of Electronics and Systems (DES), Universidade Federal de Pernambuco (UFPE), Recife 50740-550, PE, Brazil; 3 David R. Cheriton School of Computer Science, University of Waterloo, Waterloo, ON N2L 3G1, Canada

**Keywords:** EON, multipath routing, bandwidth reuse, transmission spectrum assignment (T-SA) problem

## Abstract

In this paper, we investigate a scenario in which protected and unprotected services coexist in an elastic optical network under dynamic traffic. In the investigated scenario, unprotected services can reuse the reserved idle bandwidth to provide protection to the protected services. Under this scenario, we propose a new heuristic algorithm that enables such reuse as well as define and introduce a new assignment problem in elastic optical networks, named a Transmission Spectrum Assignment (T-SA) problem. In this paper, we consider a scenario in which services may be routed using the multipath routing approach. Additionally, protection using bandwidth squeezing is also considered. We assess our proposal through simulations on three different network topologies and compare our proposal against the classical protection approach, in which bandwidth reuse is not allowed. For the simulated range of network loads, the maximum (minimum) blocking probability reduction obtained by our proposal is approximately 48% (10%) in the European topology, 46% (7%) in the NSFNET topology, and 32% (6%) in the German topology.

## 1. Introduction

Compared to Wavelength Division Multiplexing (WDM) optical networks, Elastic Optical Networks (EONs) were conceived as a more effective optical network infrastructure, as their spectral resource is divided into finer bandwidth entities (slots), and these may be provided in numbers that exactly fit the demanded traffic, instead of a rigid (single) spectrum portion as in WDM. In order to implement the service requests made to EONs, it is necessary to solve the fundamental problem of routing and spectrum assignment (RSA) [1,2,3], which consists of finding a path with a sufficiently large number of available slots that are continuous (with the same indices along the route links) and contiguous (with adjacent indices). The need to observe these two constraints might lead to a situation of misalignment between available spectral bands along the network links, which is known in the literature as spectral fragmentation (SF). SF has a deleterious effect on network performance [3,4,5,6].

During optical network operations, due to the large amount of traffic supported by an optical fiber, a link failure event would imply traffic disruption to millions of users, leading to important economic losses. Therefore, resiliency, which is the network’s ability to continue in operation when failure occurs, becomes an essential requirement to be employed in optical networks [7]. This is commonly performed by a strategy known in the literature as traffic protection [5,6,8,9], which consists of allocating, in the network, not only enough transmission capacity to meet a given service request ($B_{r}$) but also an extra capacity ($B_{i}$) that can be used in the event of failures. An alternative strategy explored in the literature for the same purpose is restoration, which differs from protection as it does not reserve resources beforehand; rather, it seeks resources only upon the occurrence of a failure. Restoration tends to consume fewer spectral resources compared to protection, but it lacks assurance for service restoration in case of failure. Additionally, successful restoration takes longer to restore the affected services compared to protection [10,11,12]. We investigate, in this paper, the utilization of a protection strategy to provide network resilience.

The classic strategy to provide protection against single-link failure is to use two link-disjoint paths with enough individual capacity to attend to the demanded request. Since the same transmission rate $B_{r}$ is allocated on both paths, it is necessary to double the total allocated capacity, i.e., $B_{t}=2B_{r}$. These paths are conventionally referred to as working (or principal) and backup (or secondary) paths. This is the well-known Dedicated Path Protection (DPP) technique, which can be directly employed in WDM or Elastic Optical Networks. The application of protection strategies can lead to excessive consumption of a network’s spectral resources (ECSRs) [5,8,9]. Therefore, enhancements to DPP have been investigated, remarkably for EONs, since the capability of allocating resources with variable granularity allows the employment of more efficient protection mechanisms [5,8,13].

An approach partially similar to DPP is Shared Backup Path Protection (SBPP), for which a working (primary) lightpath and a backup (secondary) lightpath are also pre-calculated, and their required bandwidths are reserved for each connection demand [14]. However, SBPP differs from DPP in the fact that, while in DPP backup lightpaths have their own dedicated spectrum resources, in SBPP, the resources of the backup lightpath of different demands may be shared under the condition that these resources can be used only by one demand under failure condition.

One of the key concerns of a Network Carrier (NC) is how to effectively utilize the deployed network capacity, which is related to utilizing the minimum resources to attend to new services and doing it in a coordinated way to avoid future service rejection. A form to mitigate both SF and ECSR is the use of a multipath routing strategy (MPR), which consists of spreading the total necessary transmission rate $B_{t}$ ($B_{t}=B_{r}+B_{i}$) to implement a protected service over multiple paths in the network [15,16]. If these paths are link-disjoint, the strategy is named as link-disjoint MPR (LD-MPR) [5,6,9]. When applying the LD-MPR scheme, there is no difference between working and backup paths: $B_{t}$ is divided among *P* (P>2) link-disjoint paths so that if a single-link failure occurs in one of the *P* paths, the remaining P−1 are capable of transmitting at least $B_{r}$ [8,9]. This convenient traffic partitioning not only reduces the total bandwidth required to allocate $B_{t}$ but also allows $B_{t}$ to be allocated using narrow spectral bands over multiple different paths, which enables the twofold benefit of a more effective traffic distribution among network links as well as a better fit in the existing spectral gaps and a consequent SF reduction [8,9]. There are two potential drawbacks in applying the MPR strategy: the need to allocate guard bands on each path and the differential propagation delay between these paths [15]. Another form to further mitigate ECSR is the application of bandwidth squeezing protection (BSP) [8,9]. Under a BSP regime, it is allowed that the service requested bit rate, $B_{r}$, may be reduced by, at most, a certain squeezing factor (β) under a failure in one of the links used by the service.

All the survivability mechanisms described in the references so far are designed to provide efficient reliability mechanisms for protected traffic only. However, network carriers must be prepared to attend to two kinds of customers: those who demand services that must remain active even upon failure occurrences in a network’s links and those who do not [12]. To cope with this situation, NCs may offer to their customers either a protected service (PS) or an unprotected service (US). Few works in the literature have investigated such multi-service scenarios [11,12,17]. However, none of them discuss the possibility of how unprotected connections can effectively use the spare capacity provided by protected connections when both protected and unprotected services coexist in the same network, except for the work by Lopes et al. [17] (this work is further detailed in Section 2), which addresses the opposite scenario: allowing protected services of a certain type to calculate their backup route on the same route and spectrum already used by unprotected services. In addition, the proposal by Lopes et al. cannot be applied in a multipath routing scenario because, in their work, they assume a clear distinction between the primary and backup paths, which, as discussed before, cannot be assumed in a multipath routing network.

Under the non-failure condition of an MPR protection scheme, part of the frequency slots allocated in *P* paths to cope with $B_{t}$ is being effectively used to transmit information (Tx-SLOTs), whereas another part is reserved but idle (Id-SLOTs). The Id-SLOTs are only effectively used for transmission in the case of a link failure. Any kind of spare capacity usage has a financial impact on NCs, as more services can be attended to for the same network capacity. Therefore, in order to improve the effectiveness of MPR protection mechanisms under a multi-service scenario, it is vital to discuss the possibility of how unprotected connections can effectively use the spare capacity provided by protected connections when both protected and unprotected services coexist in the same network.

In this paper, we investigate the scenario in which PS and US coexist in the network and, in addition, US can be established in the network by reusing the Id-SLOTs allocated for PSs. We use the acronym SHLRRS (services with heterogeneous levels of resilience and the reuse of idle slots) to identify this scenario. We also consider LD-MPR and BSP to deal with PSs and both single paths and LD-MPR to USs. The main contributions of this paper are the analysis of an expected practical scenario where protected and unprotected services coexist in the same network; the introduction of the problem of how spare capacity from protected services can effectively be reused by unprotected services; the definition of a new assignment problem, named Transmission Spectrum Assignment (T-SA), that arises in EONs under SHLRRS; and the proposal of a new heuristic that enables USs to reuse Id-SLOTs left by PSs. This paper introduces the problem of implementing capacity reuse by unprotected services in the spare capacity of established protected services under multipath-routed EONs assuming bandwidth squeezing capability.

## 2. Related Works

Recent studies have addressed resilience in Elastic Optical Networks by considering protection strategies through Multipath Provisioning with or without bandwidth squeezing. Takeda et al. [13] proposed a scheme to minimize the required spectrum resources necessary to provide protection in elastic optical networks. The scheme enables the allocation of different numbers of spectrum slots to each path, effectively reducing the total required spectrum resources. Using a similar approach, Assis et al. [8] proposed a mixed-integer linear programming (MILP) formulation to optimize the unequal partitioning of connections’ transmission rates among multiple link-disjoint paths to reduce total spectral usage. The authors apply the protection scheme to implement a resilient network against single-link failures and demonstrate the effectiveness of the proposed OPDPP formulation in reducing spectrum usage. Halder et al. [5] introduced the E-S-RSM-RSA scheme, optimizing spectrum and energy efficiency in regenerator-aware EONs. The proposed RSA applies the protection scheme to deal with the survivability of EONs under static traffic. Dinarte et al. [18] proposed a scheme that uses a genetic algorithm to optimize RSA ordering selection for each source–destination node pair. They introduced the Hybrid Partitioning Dedicated Path Protection (HPDPP) algorithm for resilient connection establishment with optimized R-SA or SA-R policy selection. Liu et al. [19] proposed a survivable multipath fragmentation-sensitive and fragmentation-aware routing strategy to simultaneously enhance the survivability and spectrum fragmentation of space-division multiplexing (SDM) EONs, leading to improved network blocking probability and spectrum utilization.

In terms of the coexistence of different service classes in the network, there are also recent works investigating the theme. Dixit et al. [20] and Batham et al. [21] consider three distinct classes of service (CoSs) coexisting in the network: CoS1 for real-time traffic (e.g., online audio–video traffic), CoS2 for nonreal-time traffic (e.g., compressed video and transactional data traffic), and CoS3 for delay-tolerant traffic (such as store-and-forward bulk data file transfer between various data centers). Liu et al. [22] investigate the coexistence of services with different levels of priorities, and they assumed that the priority levels can be arbitrarily defined by the network provider and its users via service level agreement (SLA). Note that in these previous three works, the assumed classes of services are differentiated either in terms of constraints regarding the delay experienced by the services or in the level of call priorities, not in terms of the level of resilience offered to each service. On the other hand, the present paper deals with different services coexisting in the network, but each service requires a different level of resilience. Hereafter, we discuss works that investigate this latter type of service differentiation.

Hai [12] analyzes the current real traffic worldwide and concludes that premium traffic (requiring protection) accounts for a portion of the total traffic, with the remaining being best-effort traffic (not requiring protection). Observing this fact, the author identifies an opportunity to optimize spectrum allocation and presents a novel integer linear programming (ILP) approach to address this scenario. To illustrate the author’s idea, let us assume a demand from node A to node B with aggregated traffic of *X* Gb/s, half composed by premium service, and the remaining being best effort. The article proposes that protection is not required for the entire *X* Gb/s flow but only for half relative to the premium service, and the author claims to address this scenario for the first time. In order to adequately address this scenario wherein service differentiation exists, the paper redesigns dedicated path protection in Elastic Optical Networks to minimize spectrum usage of resilient services.

Xiong et al. [23] and Shakouri et al. [10] investigate scenarios in which services with different resilience levels coexist in the network. Both works consider the approach known as proactive restoration, which consists of a restoration scheme that precomputes the backup path before the failure event. The frequency slots assigned to these precomputed restoration paths are “marked” but not reserved in the network. The authors assume two service classes with different resilience levels: high and low priority. Services in the high-priority class use the strategy of having precomputed restoration paths, whereas the restoration paths for low-priority class services are only computed upon the failure occurrence. It is worth noting that by using this strategy, the precomputed restoration paths may be occupied by other working paths from the high-priority class at any time, once no resources for these restoration paths are reserved as they are only marked. Each time this situation occurs, a new restoration path is calculated by the control plane to restore the restoration path that is no longer available. Additionally, the restoration paths of high-priority connections may be calculated using the resources allocated for low-priority connections. In this case, there is an optimization in the use of network slots because the same resources are being used both as working paths for low-priority services and as restoration routes for high-priority services. Upon the occurrence of a failure, the marked resources are allocated to the restoration paths of the high-priority connections, and low-priority connections, whose resources have been taken, require a restoration procedure. Notice that a new restoration path of a high-priority service is recalculated when the marked resources of its current restoration path are occupied by another working path or when one link of its current restoration path fails. The above strategy optimizes the use of resources in the network, but there is no guarantee that in the event of a failure, restoration paths will be available in the network to serve high-priority services. Note that, different from our work, which employs protection as a strategy to provide resilience to the network, Xiong et al. and Shakouri et al. employ the restoration strategy.

Lopes et al. [17] present an algorithm designed to support differentiated resilience services in SDM-EONs. The authors introduce the term quality of protection (QoP) to represent the coexistence of classes of service (CoS) with varying levels of protection guarantees. The CoS definitions are based on their QoP requirements: CoS1 exhibits high preemption and QoP (dedicated protection), CoS2 demonstrates medium preemption and QoP (shared protection), and CoS3 features no protection and no preemption (low-priority traffic). Preemption, as defined by the authors, allows for the interruption of lower-priority services in favor of higher-priority ones during the path and spectrum calculations. The algorithm proposed by the authors assumes that CoS2 services can share backup routes with other services of the same type. Additionally, the backup routes of CoS2 services may be found among the routes and spectra already allocated to CoS3 (unprotected) services without resulting in the tearing down of the active CoS3 service in the network. Lopes et al. [11] further investigated the scenario they study in [17] and proposed a new algorithm, which provides routing, resource allocation, and protection in SDM-EONs. Nevertheless, they assumed, in [11], only CoS1- and CoS3-type services, and for this reason, there is no possibility for the backup paths to reuse the spectrum already allocated for unprotected services, only preemption is allowed.

Summarizing the reviewed articles, none of them address slot reuse in an elastic optical network that employs multipath protection.In our understanding, the work closest to ours is the work by Lopes et al. [17], but as discussed at the end of Section 1 and delineated in the present section, the proposal is not applicable when considering LD-MPR under SHLRRS. Table 1 summarizes the main characteristics exhibited by each reviewed work. Additionally, we did not find any algorithm (similar to ours) in the literature that can be directly applied in the multipath-routed network and, therefore, could be used as a basis for comparison with our proposal.

## 3. Transmission Spectrum Assignment Problem

In this section, we introduce and discuss the Transmission Spectrum Assignment problem by means of an illustrative example. We begin by stating the mathematical notation. Consider a service $R_{n}$ that requires a transmission rate $B_{r}$ (in Gb/s) to be established between nodes *i* and *j* in the network using a certain type of resilience mechanism type. We denote this service by $R_{n}=\left[B_{r},type,i,j\right]$. In this paper, we consider type∈{US,PS}. Note that *P* disjoint routes ($r_{1},\ldots ,r_{p},\ldots ,r_{P}$) are required to establish a service of type PS, as well as $S_{p}$ frequency slots in each of the *P* routes. We define $a_{ps}$, where p∈{1,…,P} and $s\in \{1,\ldots ,S_{p}\}$, as the frequency index used by the *s*-th slot assigned on route *p* to a service $R_{n}$. The set of all $a_{ps}$ associated with a service $R_{n}$ can be written using the matrix $A^{\langle R_{n}\rangle }=\left\{a_{ps}\right\}$. Notice that some of the slots allocated to a PS are actually used for data transmission (Tx-SLOTs), while others are allocated/reserved (Id-SLOTs) on the network but used for a service’s data transmission only in the case of link failure. To mathematically express the assignment of these two types of slots, we use the matrix $B^{\langle R_{n}\rangle }=\left\{b_{ps}\right\}$, in which $b_{ps}=1$ if the service $R_{n}$ uses the frequency index $a_{ps}$ in route *p* as Tx-SLOT and $b_{ps}=0$ if the frequency index $a_{ps}$ is defined as Id-SLOT.

**Figure 1:**
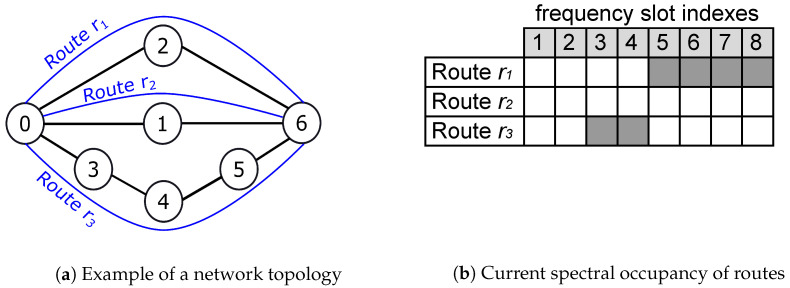
Example of a network topology with 3 link-disjoint routes (**a**) and the spectral occupancy of these routes (**b**).

Suppose the establishment of two protected services, $R_{1}\left(200,\mathrm{PS},0,6\right)$ and $R_{2}\left(200,\mathrm{PS},0,6\right)$, in the network, as shown in Figure 1. Figure 1 shows three possible link-disjoint routes to establish $R_{1}$ and $R_{2}$, as well as the current spectral occupation of each route (white and gray squares are free and occupied slots, respectively). To simplify the analysis, we assume that all three routes allow a spectral efficiency of 4 (bits/s)/Hz, and the frequency slots are 12.5 GHz wide. Thus, four slots are required to transmit 200 Gb/s. By adopting the classic 1:1 protection scheme, $R_{1}$ may be established using the routes $r_{1}$ and $r_{2}$. This choice is mathematically described by

(1) $$A^{\langle R_{1}\rangle }=\left[\begin{array}{cccc} 1 & 2 & 3 & 4\\ 1 & 2 & 3 & 4 \end{array}\right]=\left[\left(1,2,3,4\right);\left(1,2,3,4\right)\right]$$

(2) $$B^{\langle R_{1}\rangle }=\left[\begin{array}{cccc} 1 & 1 & 1 & 1\\ 0 & 0 & 0 & 0 \end{array}\right]=\left[\left(1,1,1,1\right);\left(0,0,0,0\right)\right]$$

**Figure 2:**
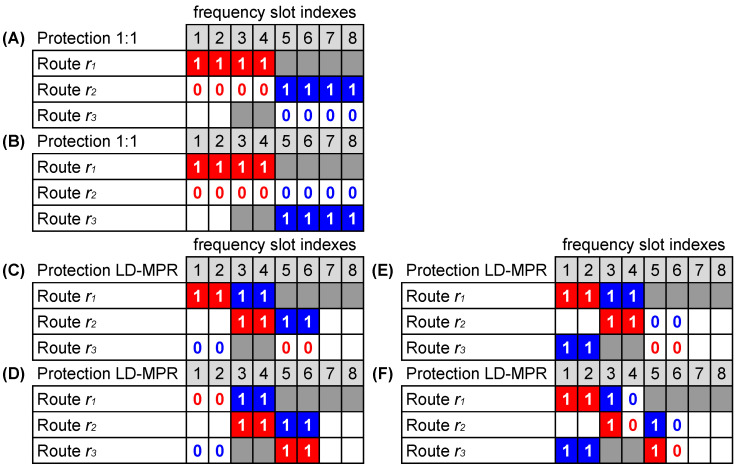
Some possibilities of solutions to the T-SA problem considering the same RSA solution and the following protection scheme: 1:1 (cases (**A**,**B**)) and LD-MPR (cases (**C**–**F**)).

Similarly, $R_{2}$ may be established using $r_{2}$ and $r_{3}$ as $A^{\langle R_{2}\rangle }=\left\{\left(5,6,7,8\right);\left(5,6,7,8\right)\right\}$ and $B^{\langle R_{2}\rangle }=\left\{\left(1,1,1,1\right);\left(0,0,0,0\right)\right\}$. Both allocations are shown in case A of Figure 2. Figure 2 shows some examples (cases A to F) of allocations that are discussed in this section. Tx-SLOTs are marked with 1 and Id-SLOTs with 0. Note that there is an alternative form to allocate Id-SLOTs and Tx-SLOTs of $R_{2}$ by calculating $B^{\langle R_{2}\rangle }=\left\{\left(0,0,0,0\right);\left(1,1,1,1\right)\right\}$, which is shown in case B.

If Id-SLOTs cannot be reused by the USs, the allocations shown in cases A and B in Figure 2 are equivalent because both RSA solutions occupy the very same routes and slot indices. However, the purpose of this paper is to investigate the SHLRRS scenario, in which USs are allowed to reuse Id-SLOTs. Under SHLRRS, for instance, in case A, it is possible to establish upcoming USs with up to four slots on $r_{2}$ and $r_{3}$ (by reusing Id-SLOTs) and with up to two slots on $r_{3}$ (using free slots), whereas in case B, it is possible to establish upcoming USs with up to eight slots on $r_{2}$ (reusing Id-SLOTs) and with up to two slots on $r_{3}$ (using free slots). Clearly, under SHLRRS, in addition to solving the RSA problem for PSs, it is also necessary to solve the T-SA. Once the routes and slots to allocate a PS are found (by RSA), the T-SA problem can be solved by assigning each of these found slots to either Tx-SLOT or Id-SLOT. In other words, while matrix *A* represents the solution of the RSA problem, matrix *B* represents the result of the solution to the T-SA problem over matrix A.

We may also analyze the allocation of $R_{1}$ and $R_{2}$ in the same scenario, but now assuming both multipath (LD-MPR) and squeezing (BSP). In this case, $B_{r}$ is equally divided among the *P* link-disjoint routes, and the bit-rate $B_{p}$ allocated for each route *p* (1≤p≤P) is given by [8,9]

(3) $$B_{p}=\frac{\left(1-\beta \right)B_{r}}{P-1},$$

in which β is the squeezing factor. For the sake of this example, we assume β=0 and P=3. For requests of $B_{r}=200$ Gb/s, we obtain $B_{p}=100$ Gb/s, which means that two slots per route are required to establish $R_{1}$ and $R_{2}$. A possible RSA solution for $R_{1}$ and $R_{2}$, under the same route states shown in Figure 1b, is $A^{\langle R_{1}\rangle }=\left\{\left(1,2\right);\left(3,4\right);\left(5,6\right)\right\}$ and $A^{\langle R_{2}\rangle }=\left\{\left(3,4\right);\left(5,6\right);\left(1,2\right)\right\}$, which is shown in Figure 2 by means of the cases C, D, E, and F. These four cases illustrate the same RSA solution but stand for different T-SA solutions. Therefore, they lead to different spectral occupations by Id-SLOTs, and as a consequence, they affect the capacity of the network differently to accommodate future USs in Id-SLOTs. This simple example shows the importance of an adequate solution for T-SA under SHLRRS.

In this article, the T-SA problem is solved using the First-Fit approach, that is, the lines of matrix *B* are successively filled with 1 s, from left to right in each line, until completing the number of 1 s necessary to establish the service.

## 4. Proposed Routing and Spectrum Assignment for Protected and Unprotected Services

In this section, we describe the proposed RSA algorithms to perform the establishment of both PSs and USs. The proposed algorithm for establishing USs enables the reuse of Id-SLOTs of PSs. Let us start by defining the mathematical notation and functions used in the routing procedures. We assume there is available an ordered set of *K* pre-computed candidate groups of *P* link-disjoint paths between each source and destination node pairs i−j, defined as $G_{P,K}^{\langle i,j\rangle }=\left\{g_{P,1}^{\langle i,j\rangle },\ldots ,g_{P,k}^{\langle i,j\rangle },\ldots ,g_{P,K}^{\langle i,j\rangle }\right\}$, in which $g_{P,k}^{\langle i,j\rangle }$ is composed of an ordered set of *P* link-disjoint routes in the network topology, i.e., $g_{P,k}^{\langle i,j\rangle }=\left\{r_{1,k}^{\langle i,j\rangle },\ldots ,r_{p,k}^{\langle i,j\rangle },\ldots ,r_{P,k}^{\langle i,j\rangle }\right\}$, where $r_{p,k}^{\langle i,j\rangle }$ and $r_{q,k}^{\langle i,j\rangle }$ are link-disjoint routes for p≠q, ∀i,j,k. The manner in which the set G is generated is described in [9]. The function SA_Reuse(*B*,*r*) performs the spectrum assignment, and it returns true if it is possible to find Id-SLOTs in route *r* capable of transmitting the bit-rate *B*. Otherwise, it returns false. Similarly, SA(*B*,*g*) performs the spectrum assignment on the routes of group *g*, and it returns true if it is possible to find free slots (Fr-SLOT) capable of transmitting the bit-rate *B* in each route that belongs to group *g*. Otherwise, it returns false. In both cases, the First-Fit algorithm is assumed to perform the spectral assignment.

Algorithm 1 is executed upon each new service request to the network. Initially, it identifies if the service is either PS or US (lines 1 and 7, respectively). If the service is US, an attempt is made to establish it using a single route by executing the function *SinglePath* (Algorithm 2), which tries to reuse the available Id-SLOTs. If this attempt is not successful, a new attempt is made (but now using two paths and no reuse) by executing the function *MultiPath* (Algorithm 3) on line 5. On the other hand, if the service is PS, we try to establish it directly using multiple paths by executing the function *MultiPath* (Algorithm 3) on line 8.
**Algorithm 1** Main($B_{r}$,*P*,*K*,*i*,*j*,type)1:**if** (type= US) **then**2:   **if** (*SinglePath*($B_{r}$,*P*,*K*,*i*,*j*) = Accepted) **then**3:     **return** Accepted;4:   **else**5:     **return** *MultiPath*($B_{r}$,2,*K*,*i*,*j*,US);6:   **end if**7:**else if** (type= PS) **then**8:   **return** *MultiPath*($B_{r}$,*P*,*K*,*i*,*j*,PS)9:**end if**

**Algorithm 2***SinglePath*($B_{r}$,*P*,*K*,*i*,*j*)
1:**for** (p=P **to** 2; p−−) **do**2:   Acquire the set $G_{p,K}^{\langle i,j\rangle }$;3:   **for** (k=1 **to** *K*; k++) **do**4:     **if** (SA_Reuse($B_{r}$,$r_{p,k}^{\langle i,j\rangle }$) = true) **then**5:       **return** Accepted;6:     **end if**7:   **end for**8:
**end for**
9:**return** Blocked;


**Algorithm 3***MultiPath*($B_{r}$,*P*,*K*,*i*,*j*,type)
1:**for** (p=P **to** 2; p−−) **do**2:   Acquire the set $G_{p,K}^{\langle i,j\rangle }$;3:   **if** (type = PS) **then**4:     $B_{p}⟵\left(\left(1-\beta \right)B_{r}\right)/\left(p-1\right)$;5:   **else if** (type= US) **then**6:     $B_{p}⟵B_{r}/p$;7:   **end if**8:   **for** (k=1 **to** *K*; k++) **do**9:     **if** (SA($B_{p}$,$g_{p,k}^{\langle i,j\rangle }$) = true) **then**10:        **if** (type = PS) **then**11:          Solve T-SA;12:        **end if**13:        **return** Accepted;14:     **end if**15:   **end for**16:
**end for**
17:**return** Blocked;


The function *SinglePath* (Algorithm 2) searches only for Id-SLOTs to establish the current service. The search is conducted by successively testing if spectrum reuse can be accomplished in the last route (loop in line 3) of each pre-computed group of *P* link-disjoint routes, $G_{p,K}^{\langle i,j\rangle }$, starting from groups with *P* link-disjoint routes to those with two routes (loop in line 1). The first route with successful spectrum reuse (function SA_Reuse in line 4) is returned as the solution to the *SinglePath* function. If none of the searched routes has enough available Id-SLOTs, the algorithm is finalized with an unsuccessful return message (“blocked” return, as shown in line 9).

The function *MultiPath* (Algorithm 3) is called either the first option of PS or for US whenever the *SinglePath* function does not succeed on its spectrum reuse exploration in the routes of the pre-computed candidate groups. *MultiPath* searches only for free slots (Fr-SLOT) to establish the current service. Loop in line 1 considers, successively, splitting the service bit-rate among *P*, P−1,…, two paths. For each possibility, its *K* pre-computed groups are acquired (line 2) and tested (loop in line 8). The bit-rate $B_{p}$ to be allocated on each of the *p* routes is calculated according to the type of current service, either PS or US (lines 4 to 6). If the service is PS, Equation (3) is employed (line 4), as stated for LD-MPR protection assignment. In case the service is US, since it does not demand traffic protection, its entire rate, $B_{r}$, is merely split among the *P* provided routes in the evaluated group (line 6). All *K* groups of link-disjoint routes are tested successively (loop in line 8) for the feasibility, or not, of allocating the current service using the *k*-th group. The tests are conducted using the function SA (line 9). In line 11, if the allocation of the service of type PS is successful, the problem of T-SA is also solved (as described in Section 3).

In a given instant of time, an Id-SLOT can be reused by only a single US. The tearing down process of a US that is reusing one or more Id-SLOTs from a PS makes these specific Id-SLOTs again available for reusing. On the other hand, the tearing down process of a PS that has active USs reusing its Id-SLOTs does not deactivate these USs, which makes them remain active in the network. In the case of a link failure, the Id-SLOTs from the PSs affected by the failure are reclaimed and the USs that were using these reclaimed slots are disconnected. Note that a link failure in an optical network is expected to be a rare event.

## 5. Simulation Setup

An elastic optical network simulator derived from SimEON [24] is used to carry out simulation results. SimEON is an open-source simulator capable of simulating the required EON characteristics relevant to the present study. SimEON and its extensions have been used and validated as a simulation engine in many works in the literature [18,25,26]. Call requests are established through a unidirectional circuit-switched lightpath. Poisson’s traffic model is assumed. The simulator generates a large number of services in each simulation. We assumed the following set up for the services: $R_{n}=\left[B_{r},type,i,j\right]$ are randomly generated considering $B_{r}\in \left\{100,200,400\right\}$ Gbits/s (uniform), type∈{PS,US} (70% of PS and 30% of US), and node pair i−j (uniform). The simulator simulates the process of setting up and tearing down services in a real network, and by doing so, it can evaluate the probability that enough resources are not available in the network to accommodate the service at its arrival time, which is referred to as the network blocking probability (BP). BP is evaluated by

(4) $$BP=\frac{\Gamma_{blocked}}{\Gamma_{accepted}+\Gamma_{blocked}},$$

in which $\Gamma_{accepted}$ and $\Gamma_{blocked}$ stands for the number of accepted and blocked services, respectively, during the simulation. The network blocking probability under this dynamic traffic is used as the performance metric. We assume P=3, K=10, β=0.2, and 320 frequency slots (each 12.5 GHz wide) available per optical fiber. We run simulations in three different network topologies: European [3], Nsfnet [5], and German [27]. We consider the 4-QAM modulation format.

## 6. Results

To study the network blocking probability reduction achieved by the proposed heuristic, we executed two different versions of it. The first, named “With Reuse”, enables USs to reuse Id-SLOTs left by PSs. The RSA problem is then solved using Algorithm 1. The second, named “No Reuse”, disables reuse capability, which is achieved using a version of Algorithm 1 obtained after the removal of lines 2, 3, 4, and 6.

**Figure 3:**
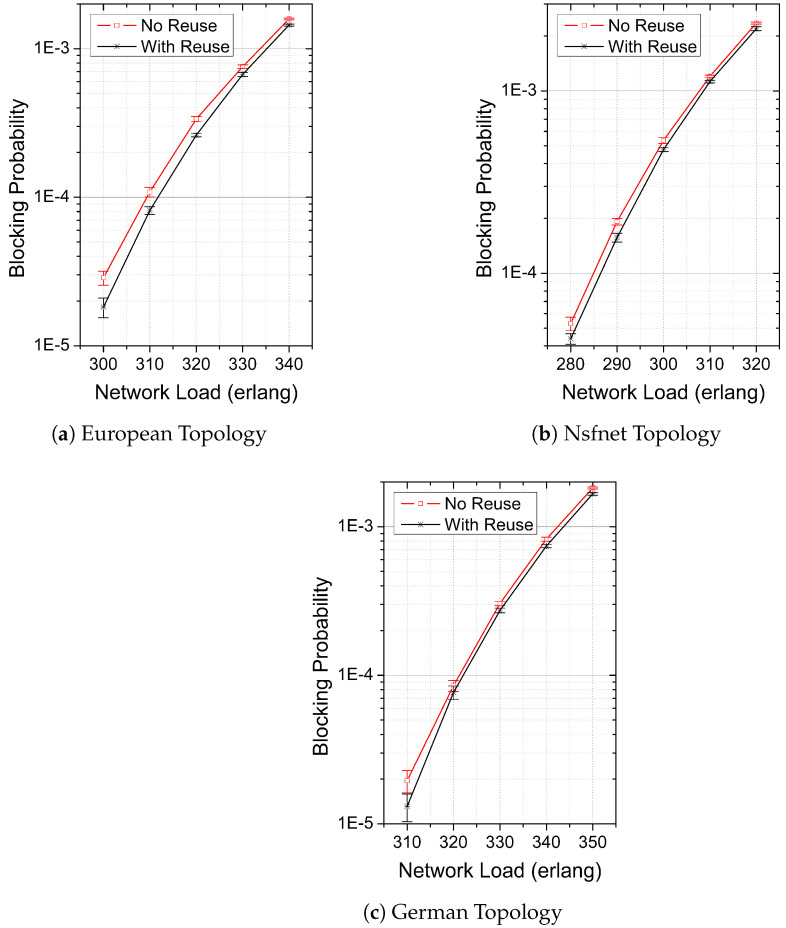
Mean values and 95% confidence intervals of blocking probabilities obtained by the “With Reuse” and “No Reuse” strategies as a function of network-offered loads in the network topologies (**a**) European, (**b**) Nsfnet, and (**c**) German.

Figure 3 shows the BP as a function of the network load for both investigated strategies “With Reuse” and “No Reuse” in the considered topologies: European (Figure 3a), Nsfnet (Figure 3b), and German (Figure 3c). The symbols plotted in the graphs stand for the mean value obtained for the BP, whereas the error bars stand for the confidence interval of 95%. We can observe that in all investigated cases, the “With Reuse” strategy achieves lower values of BP than the “No Reuse” strategy. Moreover, we observe no overlapping of the obtained confidence intervals (with the exception of 320 erlangs in the German topology), which corroborates the statistical significance of the results. For the simulated range of network loads, the maximum (minimum) BP reductions obtained by the “With Reuse” strategy in comparison to “No Reuse” are approximately 48% (10%) in the European topology, 46% (7%) in the Nsfnet topology, and 32% (6%) in the German topology.

## 7. Conclusions

In this paper, we investigated resilient Elastic Optical Networks that employ protection mechanisms under multipath routing and bandwidth squeezing in a scenario where protected and unprotected services coexist. Differently from conventional protection mechanisms, where both transmission slots and idle slots remain dedicated to the connection, we propose that unprotected services may reuse those idle slots of protected services. We identified and described, for the first time, the necessity of solving the T-SA problem in resilient networks with multipath routing where the proposed reuse is permitted.

To enable our proposal, new straightforward heuristic algorithms are presented. They deal with the spectrum reuse capability and solve the introduced Transmission Spectrum Assignment problem with the aim of properly assigning unprotected services over idle slots of protected services, mitigating the network path request blocking probability.

We examined three conventional network topologies, where we could observe that our proposal could effectively assign unprotected services to Id-SLOTs, which enabled additional traffic to be carried by the network when compared to its equivalent protection strategy without slot reuse. In the blocking probability range of approximately $10^{-5}$ to $10^{-2}$, our proposal achieved blocking probability reductions between 10% and 48% for the European topology, between 7% and 46% for the Nsfnet topology, and between 6% and 32% for the Germany topology.

By defining the efficiency of a network as the total amount of traffic capacity it can handle relative to its telecommunications infrastructure, we can state that the spectrum reuse proposal presented in this article can enhance the efficiency of a resilient EON. This is because the proposal enables unprotected services to use the Id-SLOTs, allowing more total traffic to be carried by the network. This conclusion is evidenced by the reduction in blocking probability achieved by our proposal.

We believe that more sophisticated algorithms to solve the addressed problems can lead to further reductions in the network path request blocking probability. In the case of T-SA, one way we envision achieving this reduction is by solving it in an optimized manner, using, for example, a computational intelligence algorithm. We also believe that there is still room for optimization in the routing mechanism itself, by using, for example, an adaptive routing procedure that finds the shortest path considering only the links with available Id-SLOTs.

## Figures and Tables

**Table 1:** Main characteristics exhibited by the related works found in the literature.

	[5,8,13,18,19]	[20,21,22]	[12]	[11,17]	[10,23]	This Paper
Differentiation of services		✓	✓	✓	✓	✓
Services with diff. resilience levels			✓	✓	✓	✓
Protection	✓		✓	✓		✓
Slot reuse				✓	✓	✓
Multipath (LD-MPR)	✓					✓
Bandwidth Squeezing	✓					✓

## Data Availability

Data are contained within the article.
